# Climbing ability of teneral and sclerotized adult bed bugs and assessment of adhesive properties of the exoskeletal fluid using atomic force microscopy

**DOI:** 10.1371/journal.pone.0189215

**Published:** 2017-12-15

**Authors:** Kevin R. Hinson, Vladimir Reukov, Eric P. Benson, Patricia A. Zungoli, William C. Bridges, Brittany R. Ellis, Jinbo Song

**Affiliations:** 1 Department of Plant and Environmental Sciences, Clemson University Clemson, South Carolina, United States of America; 2 Department of Bioengineering, Clemson University, Clemson, South Carolina, United States of America; 3 Department of Mathematical Sciences, Clemson University, Clemson, South Carolina, United States of America; University of North Carolina at Greensboro, UNITED STATES

## Abstract

We observed that teneral adults (<1 h post-molt) of *Cimex lectularius* L. appeared more adept at climbing a smooth surface compared to sclerotized adults. Differences in climbing ability on a smooth surface based on sclerotization status were quantified by measuring the height to which bed bugs climbed when confined within a glass vial. The average maximum height climbed by teneral (*T*) bed bugs (n = 30, height climbed = 4.69 cm) differed significantly (P< 0.01) from recently sclerotized (*RS*) bed bugs (n = 30, height climbed = 1.73 cm at ~48 h post molt), sclerotized group 1 (*S1*) bed bugs (n = 30, *S1* = 2.42 cm at >72 h), and sclerotized group 2 (*S2*) bed bugs (n = 30, height climbed = 2.64 cm at >72 h post molt). When heights from all climbing events were summed, teneral bed bugs (650.8 cm climbed) differed significantly (P< 0.01) from recently sclerotized (82 cm climbed) and sclerotized (group 1 = 104.6 cm climbed, group 2 = 107.8 cm climbed) bed bugs. These findings suggested that the external surface of teneral bed bug exoskeletons possess an adhesive property. Using atomic force microscopy (AFM), we found that adhesion force of an exoskeletal (presumably molting) fluid decreased almost five-fold from 88 to 17 nN within an hour of molting. Our findings may have implications for laboratory safety and the effectiveness of bed bug traps, barriers, and biomimetic-based adhesives.

## Introduction

The common bed bug (*Cimex lectularius* L.) has been a blood-feeding ectoparasite of humans for at least 4,000 years [1]. Despite this long-association, bed bugs were almost eliminated from the developed world in the mid-20^th^ century due to the development and widespread use of DDT [2]. However, beginning in the early 1990s, pest management professionals began seeing an increasing number of bed bug infestations throughout the country [3]. The cause of this increase is not known, but has been attributed to increased international travel, reduced use of residual insecticides indoors, insecticide resistance [4–6], lack of awareness among the general public [7], and the secondhand furniture trade [8]. Because of their cryptic nature, blood-feeding habits, and widespread insecticide resistance, bed bugs are again considered one of the most unwanted urban pests and one of the most difficult to control.

Bed bugs are unable to fly; infestations result from their hitchhiking on hosts or host items and active dispersal via crawling [9]. The ability of bed bugs to navigate their environment through active dispersal has not been well documented, particularly their ability to navigate smooth, vertical surfaces. An improved understanding of the ability of bed bugs to climb smooth surfaces could have implications for the development of effective bed bug barriers and interception devices, which are commonly used to help detect and control bed bug infestations [10, 11]. Climbing ability of bed bugs on smooth surfaces also has potential implications for laboratory safety if open-topped or poorly enclosed arenas are used for insecticide or behavior assays. Unveiling the mechanism by which bed bug tarsal or tibial structures cling to smooth surfaces also could benefit the growing field of biomimetic-based adhesives.

Our interest in bed bug climbing ability began when we observed differences in climbing ability based on the sclerotization status of individual bed bugs. Sclerotization is the hardening and stabilization of insect cuticle through the incorporation of phenolic compounds [12]. As bed bugs mature, they proceed through five instars before becoming an adult. As with all insects, proceeding to the next instar requires bed bugs to molt, or shed their exoskeleton [13]. Teneral insects are pale and soft bodied after molting, but their exoskeletons harden and darken during the sclerotization process [14]. Based on our experience with laboratory colonies, sclerotized bed bugs are more common at any given moment than are teneral bed bugs. We observed that teneral adult bed bugs (observed as being white to light yellow), although present in lower numbers, are more adept at climbing the sides of the container compared to sclerotized late instars and adults. Teneral individuals in an uncovered metal dish had to be placed in capped glass vials to prevent their escape. Teneral adults continued to climb proficiently when placed in vials.

We were unable to find references to the climbing ability of any insect based on sclerotization status. Kim et al. [15] and Hottel et al. [16] are the only other authors to evaluate bed bug climbing ability. Kim et al. [15] assessed the climbing ability of *Cimex hemipterus* (F.) and *C*. *lectularius* and found that adults of *C*. *hemipterus* were more effective climbers, presumably due to a greater number of tenant hairs on the tibial pad. Hottel et al. [16] evaluated differences in pulling forces of *C*. *lectularius* on various surfaces and their ability to climb a 45° glass surface. Studies by Kim et al. [15] and Hottel et al. [16], however, cannot explain differences in the climbing ability of bed bugs based on their sclerotization status. Tarsal structures do not change within a life stage. We, therefore, concluded that another characteristic of the teneral bed bug exoskeleton confers an adhesive property. Atomic force microscopy is one method capable of quantifying adhesive forces on such a small scale.

Atomic force microscopy (AFM) is based on the detection of small deflections of a flexible cantilever caused by the topography of a sample. Although AFM has been used to examine cell microelasticity [17,18], biomechanics [19], microrheology [20, 21], and to map cell ligands and surface receptors [22], few papers have used this method to study insects. AFM was used to study nano-physiology of the coccinellid beetle *Hippodamia convergens*, including the detection of heart beat and muscle movement [23]. Coccinellid beetles were also stimulated with light, and their responses were recorded using AFM [24]. The formation of calcium silicate and calcium phosphate nanoparticles on the body of the honeybee *Apis dorsata* Fabricius were detected [25], and *Drosophila melanogaster* Meigen wing membranes were examined for local elasticity and adhesion [26].

We developed a behavior assay that assessed the disparate climbing abilities of teneral and sclerotized adult bed bugs on a glass surface. Using atomic force microscopy, we quantified and elucidated the mechanism of the adhesive properties of their exoskeletons.

## Materials and methods

### Behavior assay

Fifth instars and adults of the “Jersey City” bed bug strain were used in this experiment. The Jersey-city strain is a moderately pyrethroid-resistant strain originally collected from Jersey City, NJ, and provided by North Carolina State University. All bed bugs were housed and fed in eight-dram vials and supplied with one strip of 1 cm x 0.5 cm manila envelope paper for harborage. Twenty, fifth-instar nymphs were housed in each of six vials, and twenty adults were housed in each of six vials. Bed bugs were not selected by sex so that an accurate representation of the population could be obtained. All vials were fitted with mesh tops secured with rubber bands to allow bed bugs to feed through the mesh on an artificial feeding system (developed by Garcia et al. [27] and modified by Montes et al. [28]). All bed bugs were allowed to feed to repletion on defribinated rabbit blood (Hemostat Laboratories, Dixon, CA) by exposing vials containing bed bugs to the artificial feeding system for ~30 minutes. All vials were maintained in a rearing room under a reversed 12:12 (L:D) photoperiod at ~40% RH and ~25°C.

All behavioral assays were conducted in the bed bug rearing room under interior, daytime fluorescent lighting, which was used for the diurnal cycle of our reversed 12:12 (L:D) photoperiod. Bed bug behavior was examined at the same humidity and temperature used in our rearing room for colony maintenance (~40% RH and ~25°C). Fifth-instar nymphs eclosed to the adult stage ~132–144 hours post-feeding. A small paintbrush was then used to transfer 10 teneral (<1 h post-molt) bed bugs to an eight-dram vial that had been washed with warm, soapy water and dried with paper towels. The exterior of this vial bore a transparent sticker ruler that spanned the height of the vial. The ruler ranged 0–7 cm from the base to the top of the vial. Once ten teneral bed bugs were transferred to the vial, climbing heights were recorded for 10 minutes. After 10 minutes of recording, vials were lightly swirled by hand for 10 seconds to re-induce activity and climbing behavior. Climbing heights were recorded for an additional 10 minutes. Climbing heights were recorded in mm, and were designated as the highest point reached by the anterior margin of the head before bed bugs fell to the bottom of the vial. Frequencies of each climbing height attained were also recorded. Bed bugs frequently climbed on the backs of others while attempting to scale the inner surface of the vial; therefore, all heights of one cm or less were not analyzed.

A total of six climbing trials were performed for the first half of each behavior assay. The first half of the behavior assay alternated between using 10 teneral adult bed bugs (referred to as *T*) and 10 sclerotized adult bed bugs (>72 h post-molt, referred to as *S1*). Therefore, the first half of an assay consisted of six trials in the order of *T*, *S1*, *T*, *S1*, *T*, and *S1*. Vials were washed with warm, soapy water and dried between each 20-minute, 10-second trial. The second half of each assay was conducted approximately 48 hours later using the same bed bugs from the first trial. New bed bugs were not used to assure that climbing ability based on sclerotization status was the factor being examined, rather than differences in climbing ability based on a particular sample of bed bugs. During this 48 hour period, bed bugs were maintained in eight-dram vials and supplied with one strip of 1 cm x 0.5 cm manila envelope paper for harborage. Bed bugs previously referred to as *T* had significantly sclerotized by this time, and were designated recently sclerotized (48 h post-molt, referred to as *RS*). All bed bugs previously referred to as *S1* were then designated *S2* (>120 h post-molt). The aforementioned materials and methods were then used to record climbing abilities of *RS*, *S2*, *RS*, *S2*, *RS*, and *S2*. This entire procedure was replicated three times, using new groups of bed bugs for each replicate. All trials were recorded using a JVC (JVC: Yokohama, Japan) Hard Disk Camcorder (model GZ-G360BU). The minimum, average, and maximum height obtained for each of the 36 (20 minute, 10 second) climbing trials was recorded. Descriptive statistics were calculated for minimum, average, maximum, and sum height climbed for *T*, *S1*, *RS*, and *S2*. A model was developed to include an effect for treatment and trial. The model was Y_ij_ = μ + Trt_i_ + Trial_j_ + ε_ij_ where Y_ij_ is the response (either the minimum, average, or maximum height obtained) in treatment i and trial j, μ is the overall mean of the response, Trt_i_ is the effect of treatment i, Trial_j_ is the random effect of trial j, and ε_ij_ is random error.

This model was analyzed to determine significance of treatment for any of the four characteristics. All analyses were conducted using SAS 9.3. Bed bug voucher specimens are deposited in the Clemson University Arthropod Collection and bear the label “Hinson dissertation, Chapter 8”.

## Results

### Behavior assay

**Fig 1:**
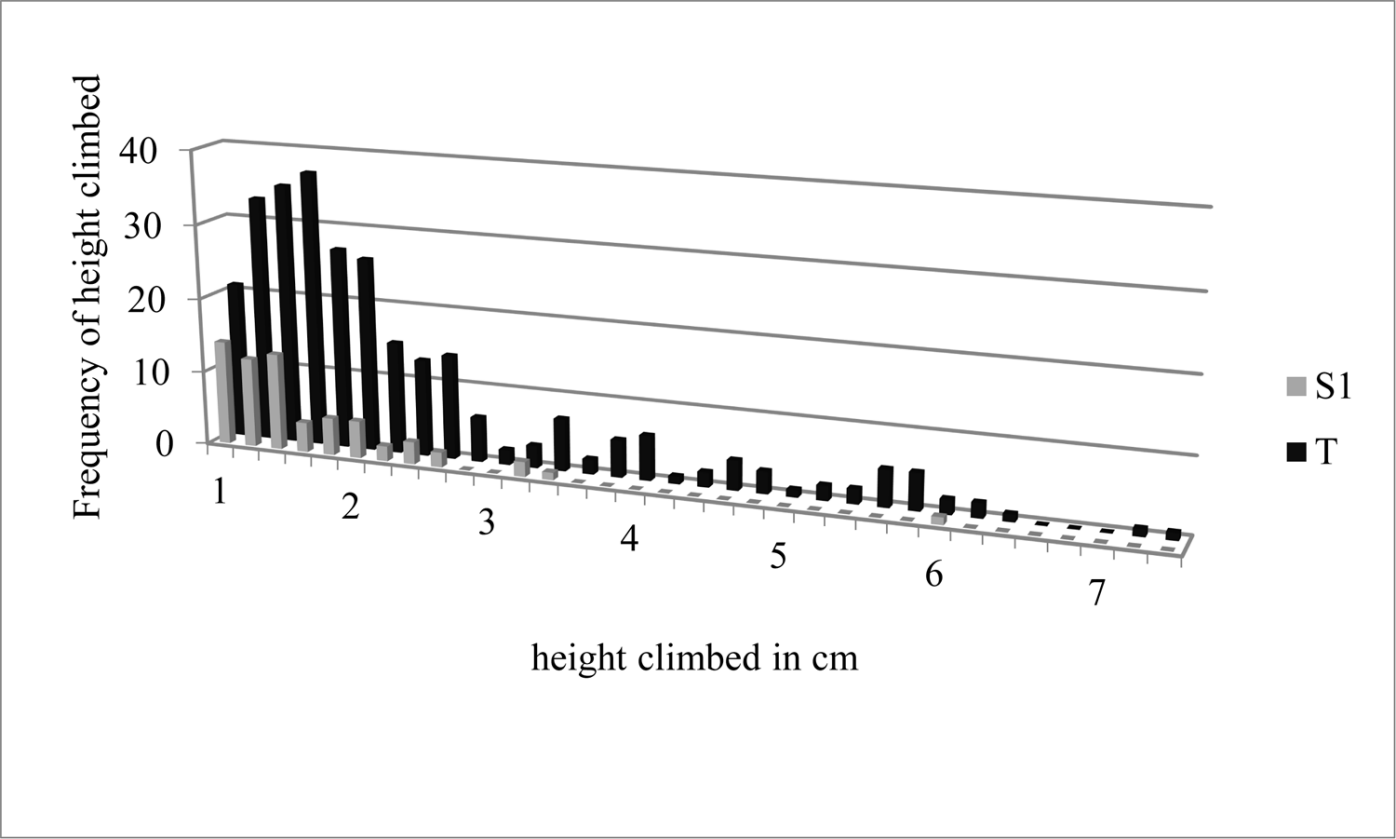
Frequencies of height climbed in cm for sclerotized adult bed bugs (*S1*) and teneral adult bed bugs (*T*) totaled across all replicates. Data used to generate Fig 1 can be found in S1 Dataset.

**Fig 2:**
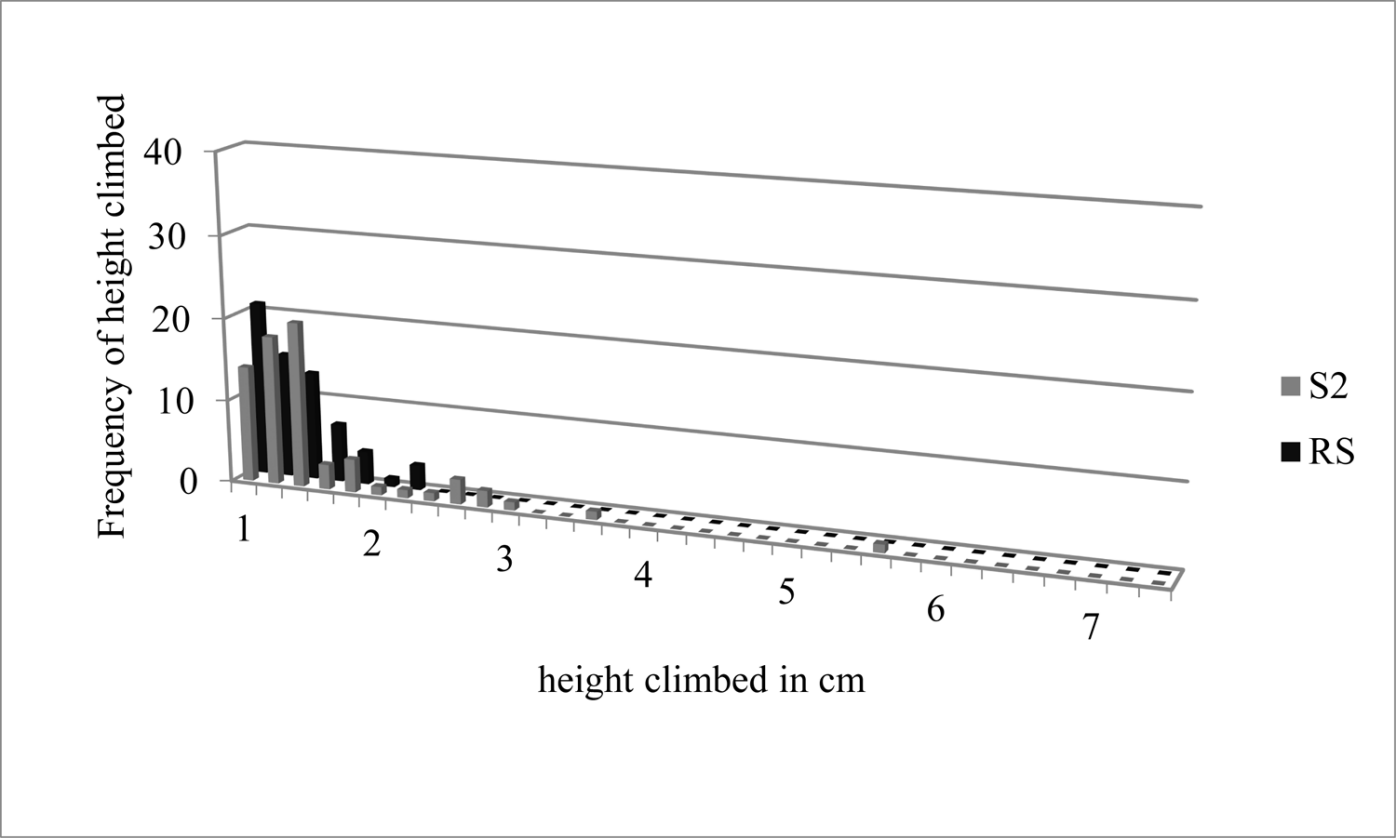
Frequencies of height climbed in cm for sclerotized adult bed bugs (*S2*) and recently sclerotized adult bed bugs (*RS*) totaled across all replicates. Data used to generate Fig 2 can be found in S1 Dataset.

**Fig 3:**
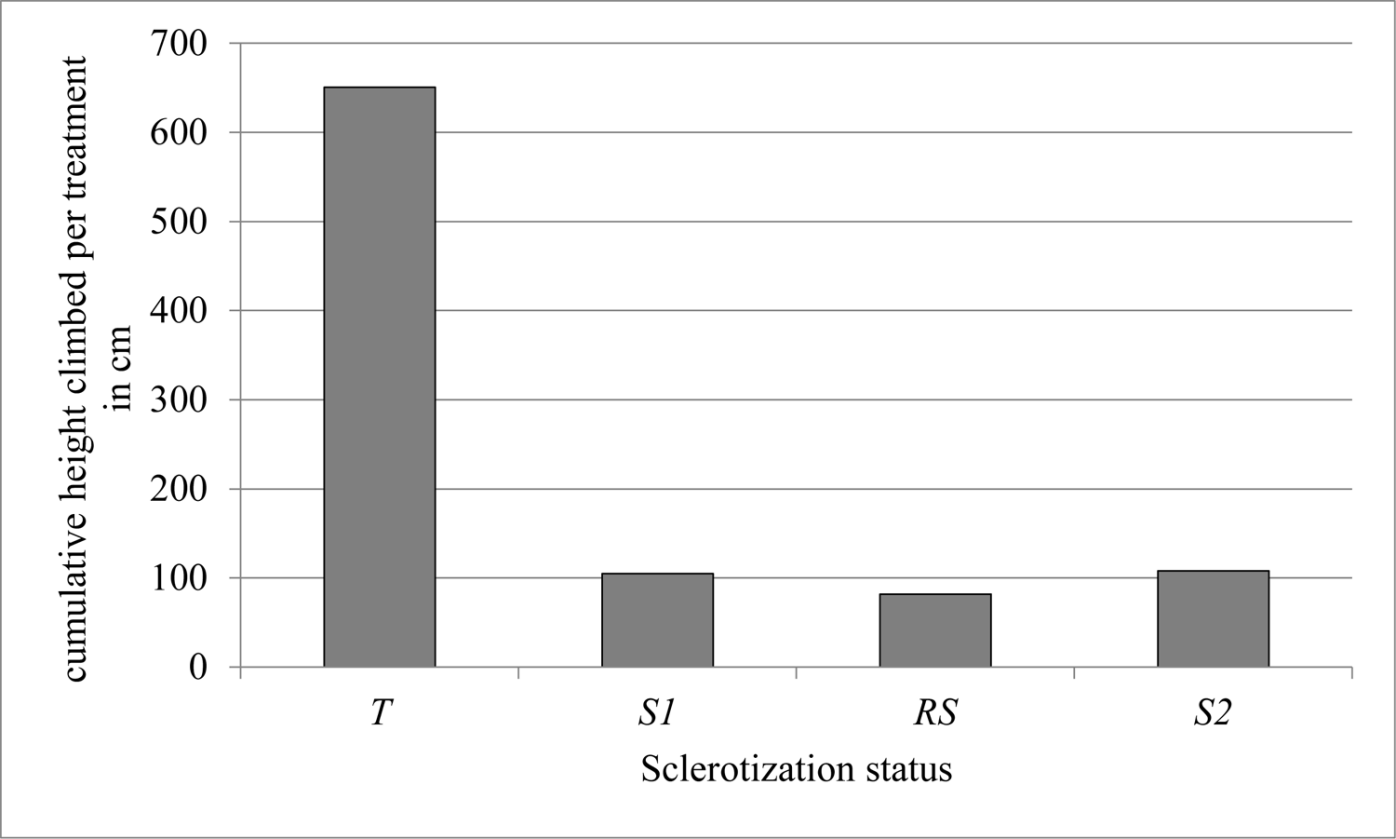
Cumulative height climbed in cm per sclerotization status totaled across all replicates. Data used to generate Fig 3 can be found in S1 Dataset.

Frequencies of climbing heights in cm were totaled for all three replicates for *T* and *S1* (Fig 1) and *RS* and *S2* (Fig 2). Centimeters climbed for all climbing events for *T*, *S1*, *RS*, and *S2* were also totaled (Fig 3).

ANOVA analyzed differences in minimum height, average height, maximum height, and cumulative height for *T*, *S1*, *RS*, and *S*2 (Table 1). Averages of minimum height, average height, maximum height, and cumulative sums were calculated, and comparisons of significant differences among molting statuses were also determined (Table 2). Comparisons between average minimum values of heights climbed were not significantly different (α = 0.05, p-value = 0.5868) (Table 1). Differences between average height climbed approached significance when comparing average values for *RS* and *T*, with *T* having the highest average height climbed among *T*, *S1*, *S2*, and *RS* (α = 0.05, p-value = 0.0932) (Tables 1 and 2). Average maximum values for height climbed were significantly different and higher for *T* compared to *S1*, *RS*, and *S*2 (α = 0.05, p-value = 0.0083) (Tables 1 and 2). Comparisons between cumulative heights climbed were also significantly different and higher for *T* compared to *S1*, *RS*, and *S*2 (α = 0.05, p-value = 0.0062) (Tables 1 and 2). The average maximum height climbed by teneral (*T*) bed bugs was higher than the average maximum height climbed by all other bed bugs (*RS*, *S1*, and *S2*), and teneral bed bugs had the highest number of total cm climbed. Given that sclerotized bed bugs attempted to climb glass vials throughout each trial but performed relatively poorly, we hypothesized that something on the surface of the teneral bed bug exoskeleton lends an adhesive property to the tibial pad or tarsi, and enables the bed bug to cling to smoother surfaces. We therefore used atomic force microscopy (AFM) to investigate the adhesive properties of bed bug exoskeletons based on sclerotization status.

**Table 1:** ANOVA output for cumulative sum and averages of minimum height, average height, and maximum height climbed in cm for *T*, *S1*, *RS*, and *S2* for all three replicates. Significant differences are indicated by asterisks(*). Data used to generate Table 1 can be found in S1 Dataset.

Treatment	Df	Sum sq	Mean sq	*F* value	*P*	Significance
minimum	3	.031	.01	.70	.587	
average	3	.973	.324	3.42	.093	
maximum	3	14.555	4.852	10.57	.008	*
cumul. sum	3	8499.107	2833.036	11.88	.006	*

**Table 2:** Cumulative sum and averages of minimum height, average height, and maximum height climbed in cm for *T*, *S1*, *RS*, and *S2* for all three replicates. LS means with the same letter are not significantly different. Statistical comparisons are within columns only. Data used to generate Table 2 can be found in S1 Dataset.

Molting status	Minimum	Average	Maximum	Cumulative sum
*T*	1.022^A^	2.089^A^	4.689^A^	650.8^A^
*S1*	1.044^A^	1.501^AB^	2.422^B^	104.6^B^
*RS*	1.156^A^	1.352^B^	1.733^B^	82^B^
*S2*	1.06666667^A^	1.4815751^AB^	2.64444444^B^	107.8^B^

## Materials and methods

### Atomic force microscopy

**Fig 4:**
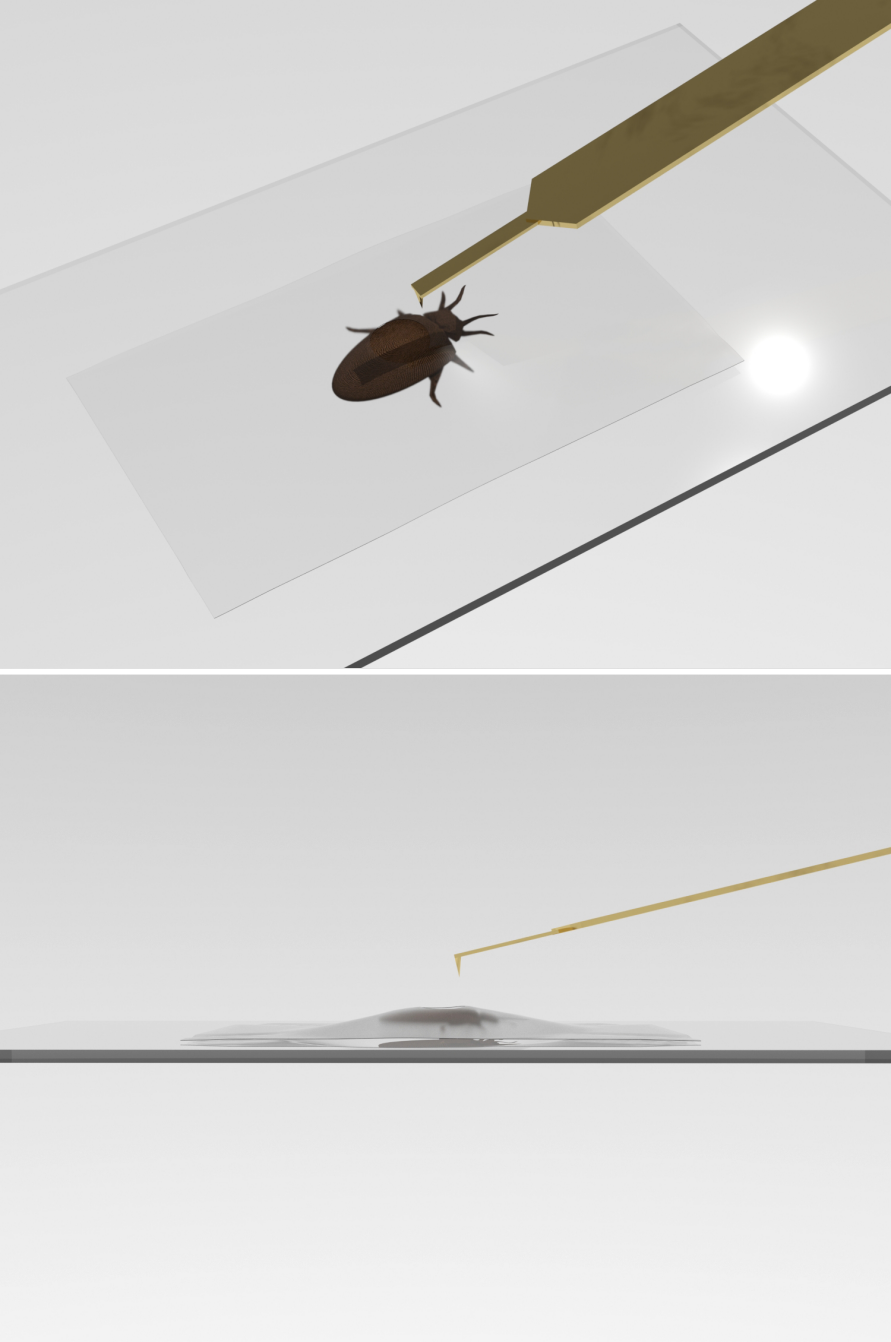
Diagram displaying lateral and dorsolateral view of a bed bug fixed on a glass slide with Scotch tape with an opening for adhesive property measurements.

To assess the adhesive property of fluid on the exoskeleton, bed bugs were immobilized on glass slides, using Scotch tape with a small opening in the tape to permit dorsal abdominal contact for AFM measurements (Fig 4). Measurements were taken using an atomic force microscope (Asylum BIO-MFP 3D) with silicon nitride Olympus TR400PB cantilevers (gold-coated, pyramidal shaped tips with a nominal tip radius of 30 nm and a resonance frequency of 32 kHz). Cantilevers had a spring constant of ~0.09 N/m and were calibrated prior to measurements of each bed bug. New cantilevers were used for each bed bug. To determine adhesive properties, a series of force-distance curves were recorded in contact mode. Multiple curves were recorded for each bed bug due to high rejection rate because of bed bug movement. The rate of approach and retraction was chosen to be 1μm/s. A total of five bed bugs were used for data analysis. In-house MatLAB code was used to analyze data.

Force-mapping approach also was used to capture representative images of bed bug adhesiveness. Acquisition of force curves can be automated when data are collected over a designated area. After generating a force map, we plotted an overlay of a 2D adhesion force map over a 3D reconstruction of bed bug topography.

## Results

### Atomic force microscopy

**Fig 5:**
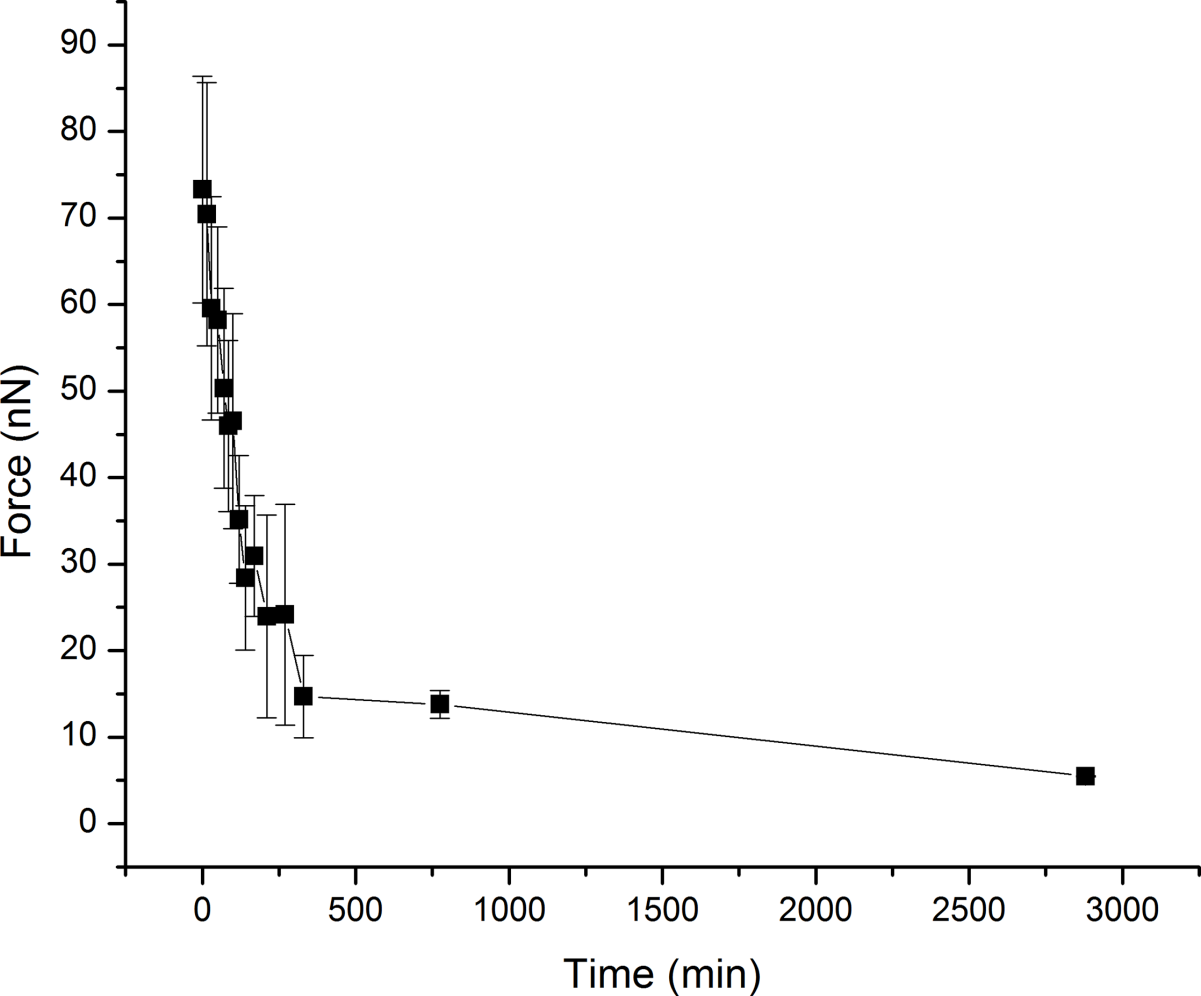
Change in adhesion force in nN measured over time after molting to adulthood. Error bars represent standard deviation.

**Fig 6:**
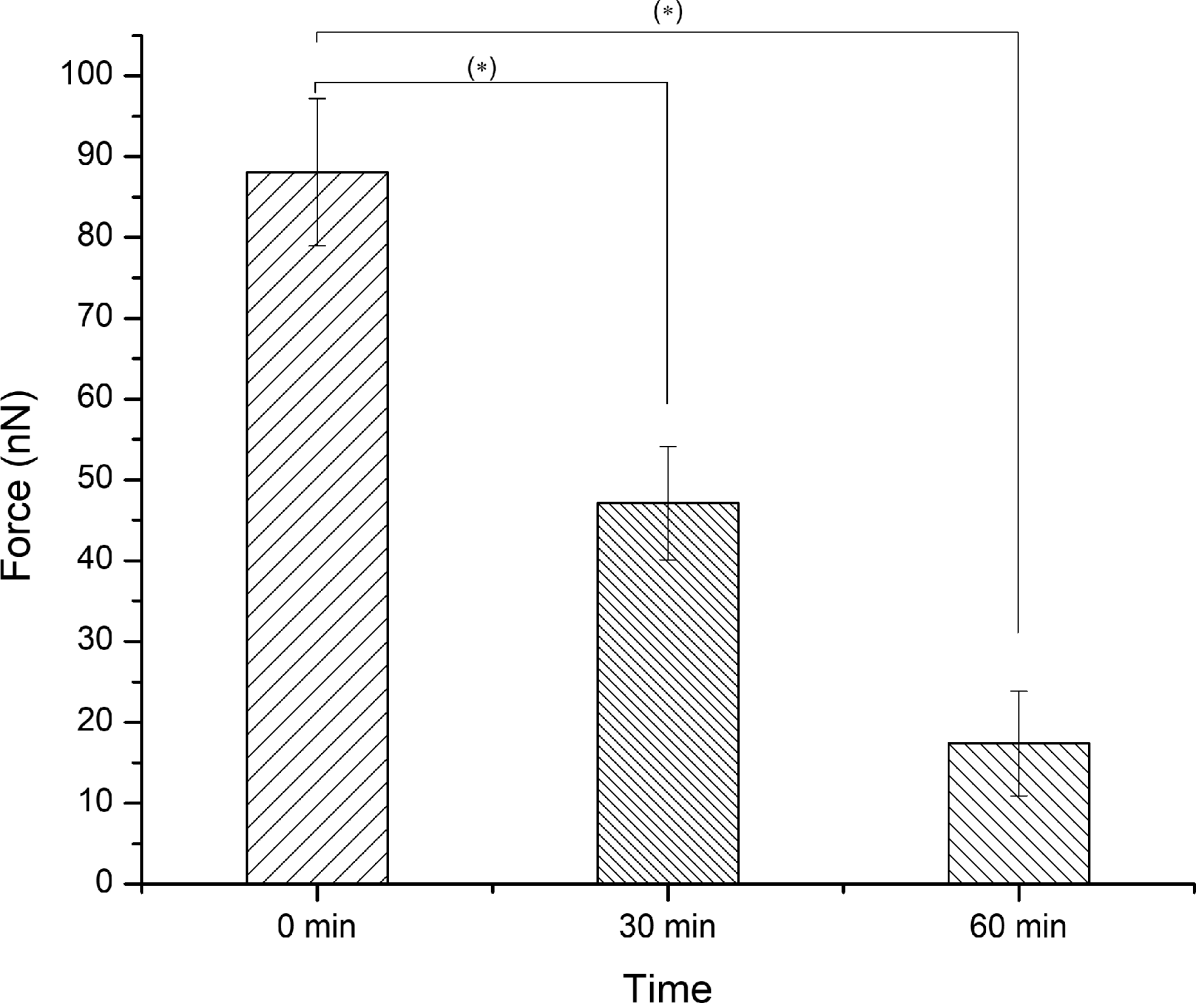
Adhesiveness of exoskeletal fluid measured in nN of force required to pull the probe cantilever from the surface of the bed bug exoskeleton. Error bars represent standard deviation. Comparisons significant at the 0.05 level are indicated by (*).

**Fig 7:**
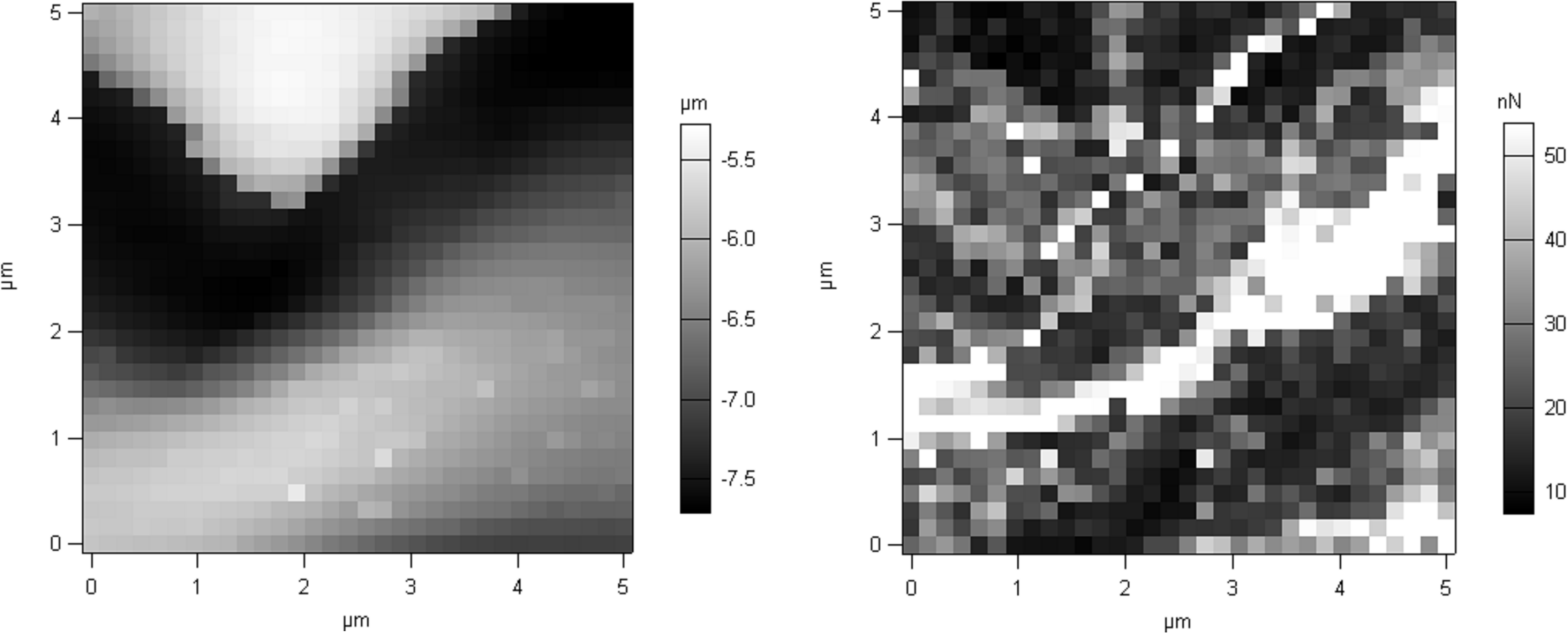
Maps generated from measurements taken of the teneral bed bug exoskeleton. The left image displays topography; the right image displays adhesion forces. Data used to generate Fig 7 can be found in S2 Dataset.

**Fig 8:**
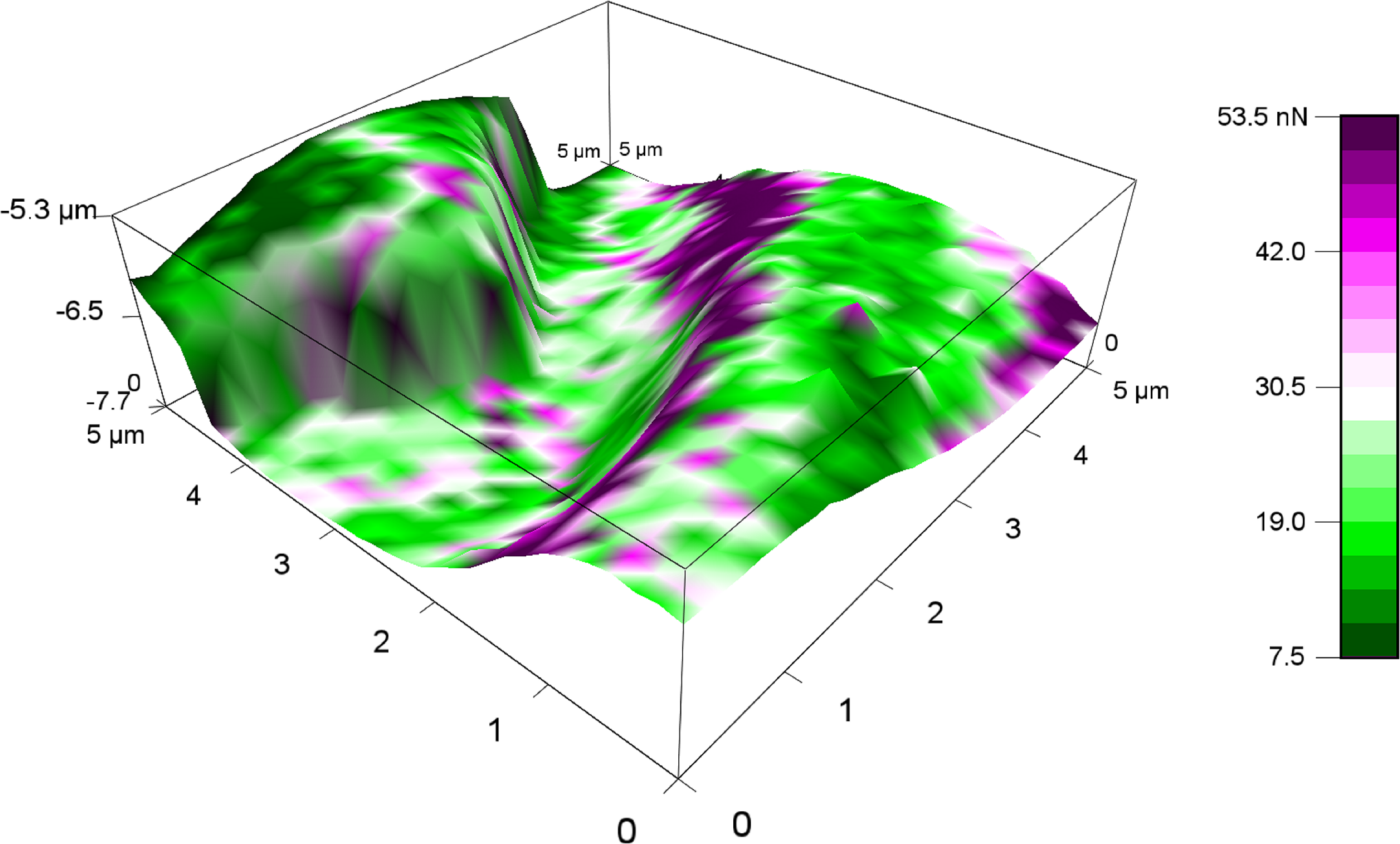
An overlay of 2D force map over 3D topography image performed on a teneral bed bug. The color scale bar displays adhesion forces. Data used to generate Fig 8 can be found in S2 Dataset.

Measurement of adhesive force was conducted for 48 hours (Fig 5). Newly molted bed bugs showed high attractive forces toward the tip of the cantilever. Change in adhesion force was most pronounced within the first hour of molting (Fig 5). In light of this, we determined average mean forces of adhesion within the first hour of molting. Adhesion force values were 88±9 nN for freshly molted bed bugs, 47±7 nN for ~30 min post molt bed bugs, and 17±6 nN for 1 hour post molt bed bugs (Fig 6). One-way ANOVA was used to confirm statistical significance between group means. Acquisition of force curves are also displayed (Fig 7). Our overlay of a 2D adhesion force map over a 3D reconstruction of bed bug topography (Fig 8) demonstrated that adhesion forces were fairly constant on the outer parts of the bed bug and slightly higher in the caverns, where it took longer for fluid to solidify.

## Discussion

Teneral bed bugs differed significantly in maximum and cumulative height climbed, and approached significance for average height climbed when compared with sclerotized bed bugs. As *RS* performed similarly to *S1* and *S2*, teneral bed bugs demonstrated superior climbing abilities. Although bed bugs are generally inefficient climbers, teneral bed bugs might possess softer and more pliable tarsal or tibial structures that could aid in climbing. The structures used by *C*. *lectularius* when climbing smooth surfaces have not been conclusively demonstrated. Although Wigglesworth [29] claimed that the tibial pad is not used by *C*. *lectularius*, Kim et al. [15] reported that *C*. *lectularius* and *C*. *hemipterus* use the tibial pad.

Regardless of the tibial or tarsal structures used, we suggest that an exoskeletal fluid enables teneral bed bugs to cling to smoother surfaces. We suggest that this fluid probably is residual molting fluid. The release of molting fluid between the insect epidermis and the old exoskeleton prior to molting supports our hypothesis. Molting fluid contains enzymes that digest the old endocuticle but does not affect the old exo- or epicuticle [14]. The digestive products are then resorbed into the body as the new cuticle is being deposited [14]. The surface of this new cuticle might contain residual digestive products (molting fluid and/or degraded cuticle) that give tarsal or tibial structures an adhesive quality. The possibility that this fluid has adhesive properties is supported by the biochemical composition of insect cuticle, which consists of chitin embedded in a protein matrix. Chitin is primarily composed of monomers of the sugar N-acetylglucosamine [14]. Chitin is reduced to N-acetylglucosamine via chitose and chitobiose, which is present in molting fluid [30]. The sugar N-acetylglucosamine may lend an adhesive property to the exoskeleton, which diminishes as the water component of the fluid evaporates from the surface of the exoskeleton. Cement and/or wax layers may have some role in adhesion, but regionalized pooling of fluids observed in this study is more indicative of a liquid. Although the adhesive property of this fluid is significantly less soon after molting, it is unknown whether this translates to a loss in climbing ability. Future researchers might examine the chemical composition of this adhesive fluid and determine if bed bugs lose climbing ability in less than 48 hours.

The ability of sclerotized bed bugs to climb glass, and the tendency for teneral bed bugs to climb to greater heights, suggests that caution needs to be taken when working with bed bugs in an open environment or designing experiments that house bed bugs in seemingly inescapable containers. The need for more secure enclosures may be particularly important if repellency testing is involved; such testing might allow recently molted bed bugs to escape containers. These findings might also have implications for the design of intercept traps and bed bug monitors. If products are designed to retain bed bugs or prevent them from crossing a barrier, such products may not be entirely effective if they are based on the assumption that bed bugs cannot climb smooth surfaces.

Our study evaluated the climbing ability of teneral and sclerotized bed bugs in a glass vial. Under natural ecological conditions (within mattress seams, bed frames, etc.) teneral bed bugs may avoid other bed bugs and remain quiescent until sclerotization is complete. Nonetheless, we have observed intercept traps containing many more exuviae than adult bed bugs. Bed bugs temporarily captured by such devices are also in an unnatural ecological condition, where hungry, sclerotized bed bugs may repeatedly attempt to escape interception devices. As shown in our experiments with glass vials, the activity of sclerotized bed bugs may disrupt the quiescent behavior of teneral bed bugs and encourage climbing. Our observation of unusual numbers of exuvia present in interception devices is only anecdotal evidence of their capacity to escape such devices in a field setting, therefore, further quantifiable observations of teneral bed bug behavior in the field is necessary to determine whether sclerotization status affects bed bug barrier or interception device efficacy. If it can be demonstrated that teneral bed bugs pose a threat to interception device efficacy, examining the climbing ability of teneral bed bugs in the presence of a desiccant might reveal that climbing can be inhibited by interfering with the adhesive nature of the teneral bed bug exoskeleton.

We have presented a novel use of AFM technology by examining adhesive fluids on the bed bug exoskeleton, but this new application did not come without some unexpected difficulties. As forces were measured on a nano scale, slight movements of the object examined (in this case, a living insect) interfered with measurements. Keeping a living insect stationary for measurements proved difficult for force mapping, which requires extended time to collect data. Although we observed molting fluid on the tarsi of bed bugs and preferred to evaluate the adhesive properties of this structure over time, bed bug tarsi moved too frequently to permit reliable measurements over time. Even when evaluating the adhesive nature of the bed bug dorsum, many trials had to be abandoned once it became clear that bed bugs were ineffectively restrained and generating erratic measurements. Although the methods we used to restrain bed bugs represented improvements, more effective methods might be devised. Future researchers may choose to experiment with alternatives, such as exposing insects to low-oxygen environments to reduce activity and facilitate nanoscale measurements.

More than 1,000,000 species of insects have been described [31]. Thus, it is surprising that no other research based on molting status has been conducted on insect climbing ability. Our findings potentially extend to other taxa, including other species of economic importance. Researchers might investigate whether this phenomenon is present across juvenile stages or differs between males and females. Additional studies may examine the exact structures used by *C*. *lectularius* when climbing smooth and rough surfaces, how rapidly these structures sclerotize, and whether the removal of exoskeletal fluid affects climbing ability.

## Supporting information

S1 Dataset Excel data used to generate Figs 1–3. (XLSX)

S2 Dataset AFM data used to generate Figs 7 and 8. (ARDF)
